# Surgical Resection of a Solitary Metachronous Retrocaval Lymph Node Metastasis above the Renal Vein from Rectal Cancer

**DOI:** 10.70352/scrj.cr.25-0325

**Published:** 2025-07-18

**Authors:** Yui Sawa, Yoshihiro Ono, Manabu Takamatsu, Gaku Shimane, Hayato Baba, Ryota Ito, Kota Sugiura, Yoshiyuki Shibata, Kaoru Nakano, Toshiki Mukai, Kosuke Kobayashi, Atsushi Oba, Yosuke Inoue, Hiromichi Ito, Takashi Akiyoshi, Yu Takahashi

**Affiliations:** 1 Division of Hepato-Biliary-Pancreatic Surgery, Cancer Institute Hospital, Japanese Foundation for Cancer Research, Koto-ku, Tokyo, Japan; 2 Department of Pathology, Cancer Institute Hospital of the Japanese Foundation for Cancer Research, Koto-ku, Tokyo, Japan; 3 Department of Colorectal Surgery, Cancer Institute Hospital, Japanese Foundation for Cancer Research, Koto-ku, Tokyo, Japan

**Keywords:** retrocaval lymph node, postcaval lymph node, para-aortic lymph node, colorectal cancer metastasis, inferior vena cava

## Abstract

**INTRODUCTION:**

Colorectal cancer (CRC) can metastasize to various sites, including the liver, lungs, ovaries, adrenal glands, and lymph nodes. Approximately 1%–2% of patients with CRC develop para-aortic lymph node metastases. Herein, we report a case of surgical resection of an isolated, metachronous, retrocaval lymph node recurrence of rectal cancer above the renal vein.

**CASE PRESENTATION:**

A 77-year-old woman was diagnosed with a solitary metachronous retrocaval lymph node metastasis from rectal cancer. The patient underwent robot-assisted laparoscopic abdominoperineal resection. The pathological status was T3, N0, M0, or Stage IIA. No recurrence was observed for 13 months after the initial surgery. CT revealed a 24-mm tumor on the dorsal side of the inferior vena cava (IVC). Tumor resection was performed, including right adrenalectomy, Spiegel lobectomy, and partial resection of the IVC. Pathological findings revealed adenocarcinoma metastasis to a solitary lymph node, which invaded the IVC and Spiegel lobe.

**CONCLUSIONS:**

This is the first report of a surgical resection of a retrocaval lymph node metastasis from CRC that invaded both the IVC wall and the liver.

## Abbreviations

CEA
: carcinoembryonic antigen
CRC
: colorectal cancer
IVC
: inferior vena cava

## INTRODUCTION

CRC is the third most common cancer and the second leading cause of cancer-related deaths in the world.^1)^ CRC can metastasize to various sites, including the liver, lungs, ovaries, adrenal glands, and lymph nodes.^2)^ Approximately 1%–2% of patients with CRC develop para-aortic lymph node metastases.^3)^ However, currently no studies have reported metastases from CRC to the retrocaval lymph nodes above the renal vein. Herein, we report a case of surgical resection of an isolated metachronous retrocaval lymph node recurrence of rectal cancer above the renal vein.

## CASE PRESENTATION

**Fig. 1:**
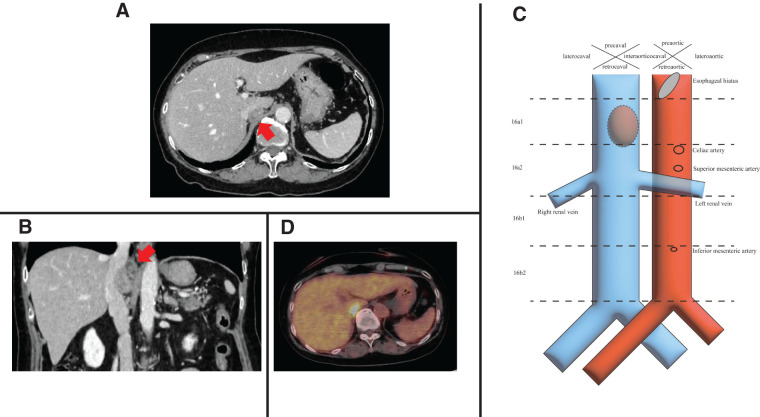
Preoperative imaging findings. (**A**) CT showed a 24-mm size tumor in the retrocaval area (red arrow). (**B**) CT showed the retrocaval tumor attached to the posterior wall of the IVC, and invasion of the IVC wall was suspected (red arrow). (**C**) If this tumor was a lymph node, it was classified as a 16a1 retrocaval lymph node according to the classification of Regional Lymph Nodes of the Japan Society of Clinical Oncology. (**D**) PET showed high fluorodeoxyglucose uptake in the tumor (standardized uptake value max: 9.23). CT, computed tomography; IVC, inferior vena cava; PET, positron-emission tomography

A 77-year-old woman was diagnosed with a solitary metachronous retrocaval lymph node metastasis from rectal cancer. She was diagnosed with rectal cancer (cT3 N1 M0, and Stage IIA), presenting with bloody stools and anal pain in 2023. After laparoscopic colostomy, the patient underwent short-course radiotherapy (5 × 5 Gy). The patient then underwent robot-assisted laparoscopic abdominoperineal resection. The pathological findings revealed a type 2 rectal tumor measuring 70 × 50 mm, diagnosed as adenocarcinoma (tub2 > tub1). The pathological stage was ypT3, N0 (0/28), with moderate venous invasion, ypStage IIA in the Japanese Classification of Colorectal, Appendiceal, and Anal Carcinoma,^4)^ and ypT3, N0, ypStage IIA in the TNM classification of malignant tumors.^5)^ Both the distal and radial margins were negative. The patient refused to undergo adjuvant chemotherapy. The patient was regularly followed up after surgical resection of the primary tumor. No recurrence was observed for 13 months after the initial surgery. CT revealed a 24-mm tumor on the dorsal side of the IVC between the confluence of the 3 hepatic veins and the left renal vein (**Fig. 1A**, **1B** and **Supplementary Video 1**). Lymph nodes in this location were classified as a 16a1 retrocaval lymph node, based on the classification of Regional Lymph Nodes of the Japan Society of Clinical Oncology (**Fig. 1C**).^6)^ PET revealed high fluorodeoxyglucose uptake in the tumor (standardized uptake value max: 9.23) (**Fig. 1D**). Metachronous solitary retrocaval lymph node metastasis from the rectal cancer was suspected. Typically, chemotherapy is administered first, and surgical resection is considered only if it is proven effective and no other distant metastases are identified. However, in this case, the tumor was located deep within the body, making it difficult to perform a biopsy and confirm whether the retrocaval tumor originated from CRC. Therefore, surgical resection was performed as the initial treatment. Her height and weight were 149.6 cm and 47.4 kg, respectively. Tumor markers were within normal ranges (CEA: 4.1 ng/mL; carbohydrate antigen: 19–9:18.4 U/mL), and no abnormalities were observed in other blood test results (hemoglobin: 12.6 g/dL; leukocytes: 6970/μL; C-reactive protein: 0.13 mg/dL; creatinine: 0.67 mg/dL; aspartate aminotransferase: 23 U/L; alanine aminotransferase: 12 U/L; total bilirubin: 0.3 mg/dL; platelets: 228000/μL; prothrombin time international normalized ratio: 0.90; hemoglobin A1c: 6.5%). She had a history of hypertension and had undergone surgery for a left femoral neck fracture. The American Society of Anesthesiologists Physical Status Classification System score was 2. Preoperative CT showed that the tumor was in contact with the IVC, Spiegel lobe of the liver, and right adrenal gland (**Supplementary Video 1**), and tumor invasion of these organs was suspected.

**Fig. 2:**
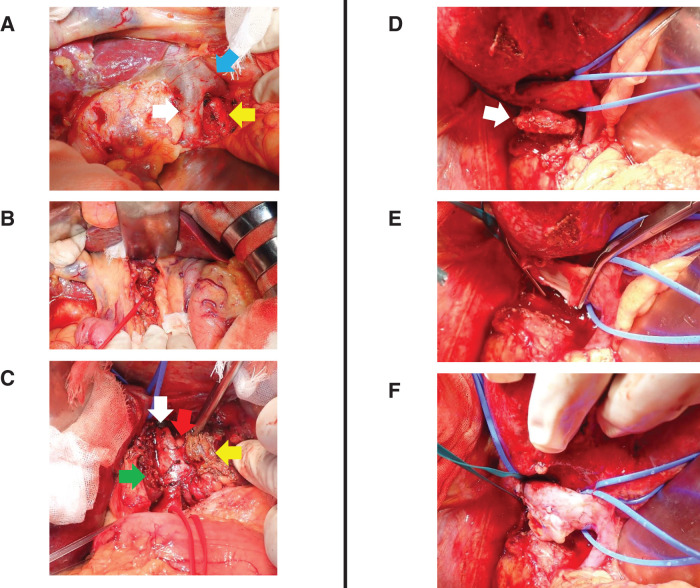
Intraoperative findings. (**A**) After sampling the laterocaval, precaval, interaortocaval, and preaortic lymph nodes below the renal vein, the IVC (white arrow), left renal vein (blue arrow), and descending aorta (yellow arrow) were identified. (**B**) After sampling the #8a lymph node, the common hepatic artery was taped with a red tape. (**C**) After transection of the Spiegel lobe, the liver was divided into the remnant segment 1 (green arrow) and the resected Spiegel lobe (yellow arrow) in front of the IVC (white arrow). The tumor was located between the IVC and resected the Spiegel lobe (red arrow). (**D**) After resection of the Spiegel lobe, partial resection of the right adrenal gland, and dissection from the diaphragm, the retrocaval tumor was attached to the posterior wall of the IVC (white arrow). The IVC and inferior right hepatic vein were taped with blue tapes. (**E**) After resection of the tumor, there was a defect in the posterior IVC wall. (**F**) Patch reconstruction with a round ligament was performed. IVC, inferior vena cava

**Fig. 3:**
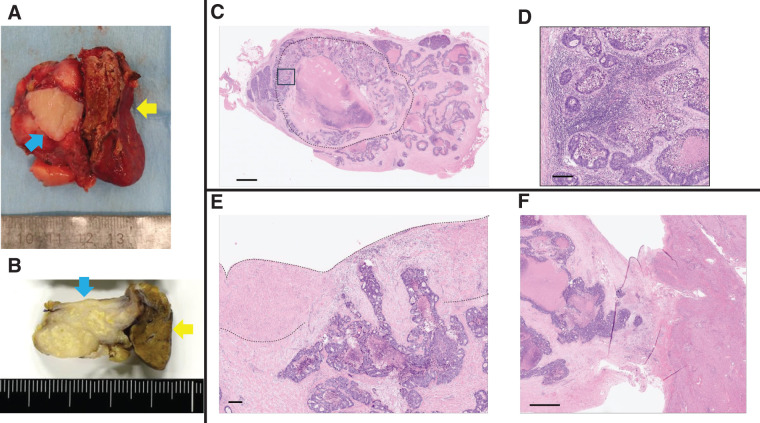
Histological findings. (**A**) The tumor was resected with the IVC wall (blue arrow) and Spiegel lobe (yellow arrow). (**B**) The specimen was sliced along the coronal section. The tumor invaded the IVC (blue arrow) and Spiegel lobe (yellow arrow). (**C**) Whole section of metastatic cancer with a dashed line indicating the presumed lymph node area. The square corresponds to (**D**). (**D**) Magnified image of cancer at the lymph node periphery, where lymphocytes are aggregating along the edge. (**E**) Cancer cells at the metastatic site invaded the IVC (dashed line). (**F**) Cancer cells at the metastatic site invaded the liver. Scale bars are (**C**) 2 mm, (**D**) 200 μm, (**E**) 200 μm, and (**F**) 1 mm. IVC, inferior vena cava

Tumor resection was performed, including right adrenalectomy, Spiegel lobectomy, and partial resection of the IVC (**Supplementary Video 2**). The surgical details are described below. After confirming the absence of liver or peritoneal metastases and a negative result on peritoneal washing cytology, Kocher mobilization was performed. Frozen section analysis revealed no lymph node metastasis at the laterocaval, precaval, interaortocaval, or preaortic (lymph node stations 16a and 16b), or lymph node stations 8a and 9 (**Fig. 2A**, **2B**). Mobilization of the right and left liver was performed. Tumor invasion of the IVC, Spiegel lobe, and right adrenal gland was suspected, and partial resection of the right adrenal gland and Spiegel lobectomy were performed (**Fig. 2C**, **2D**). Vascular occlusion was performed from the caudal side of the confluence of the 3 hepatic veins to the cranial side of the inferior right hepatic vein. Finally, tumor resection with wedge resection of the IVC was performed, and the IVC defect was reconstructed using a round ligament (**Fig. 2E**, **2F**). The operative time was 319 min, and the estimated blood loss was 330 mL. The postoperative course was good, although the patient developed an upper respiratory tract infection. The patient was discharged on POD 16. Hematoxylin and eosin staining of the histological specimens revealed an adenocarcinoma component with lymphocytes. She was diagnosed with a solitary lymph node metastasis from rectal cancer that had invaded the IVC and Spiegel lobe (**Fig. 3**). The patient received adjuvant chemotherapy and underwent regular postoperative checkups.

## DISCUSSION

Herein, we report a case of surgical resection of an isolated metachronous retrocaval lymph node recurrence of rectal cancer above the renal vein, along with a review of the previous literature. This solitary recurrence at this location has not been previously reported.

Distant metastases from CRC can be treated by surgical resection, which improves prognosis and aims for a complete cure.^7)^ Patients who undergo complete resection of liver metastases have a 5-year survival rate of approximately 40%.^8)^ Surgical resection of lung metastases can achieve a 5-year overall survival rate of 40%–68%.^9)^

The 5-year survival rate of patients with synchronous para-aortic lymph nodes who underwent para-aortic lymph node dissection is 37.7%.^10)^ In contrast, the 5-year survival rate of patients with isolated para-aortic lymph nodes who underwent para-aortic lymph node dissection is 61.1%, and the median overall survival (OS) was 34–80 months.^11,12)^ Significant factors for survival include the number of metastatic para-aortic lymph nodes, absence of other distant metastases, and a preoperative CEA level <5 ng/mL.^3,11)^ This patient had some good prognostic factors, including metachronous metastasis, a solitary lymph node, no other distant metastases, and a low CEA level, suggesting that long-term survival could be expected.

In typical cases of rectal cancer, para-aortic lymph node metastasis tends to occur initially in the lymph nodes located below the renal veins, starting from the lymph nodes around the root of the inferior mesenteric artery and subsequently spreading upward.^13)^ The para-aortic lymph nodes are interconnected anteriorly, posteriorly, and laterally, but lymphatic structures are sparse on the dorsal side of the IVC above the renal vein.^14)^ In addition to the major lymphatic vessel draining into the thoracic duct through the aortic hiatus, there may be thin lymphatic vessels that run medial and lateral to the crus of the aorta.^15)^ In this case, it is presumed that the cancer cells traveled from the rectum through the root of the inferior mesenteric artery, entered the para-aortic region, and ascended along the interconnected pathways without being captured by intervening lymph nodes, reaching the site of the metastatic lymph node. Since the cancer cells would have to pass through multiple lymph node stations without being trapped at each point, such a pattern of metastasis is considered extremely rare. The literature includes 1 report on the metastatic location of para-aortic lymph nodes from CRC^16)^; however, reports of metastases from CRC to the retrocaval lymph nodes are lacking. There has been only 1 case report of a retrocaval lymph node below the renal vein from renal cell carcinoma.^17)^ In a study that investigated the lymphatic pathways in cervical cancer, no retrocaval lymph node metastasis was observed above the level of the renal vein, whereas some retrocaval lymph node metastases were observed below the level of the renal vein.^18)^ In fact, during para-aortic lymph node dissection, the retrocaval area is usually not included within the standard dissection field.^13,19)^

In the present case, owing to the rare pattern of recurrence, differential diagnosis was required. Among these, retroperitoneal tumors, malignant lymphoma, and inflammatory diseases, such as immunoglobulin G4-related diseases, were considered. This highlights the need for excisional biopsy for treatment and to achieve a definitive diagnosis. In the present case, the retrocaval lymph node metastasis had invaded both the IVC wall and liver and required resection of these structures.

## CONCLUSIONS

This report is the first to present the surgical resection of a retrocaval lymph node metastasis from CRC that invaded both the IVC wall and the liver.

## DECLARATIONS

### Funding

The authors received no specific funding for this work.

### Authors’ contribution

Y.Sa. performed the surgical procedures, data interpretation, and preparation of the manuscript.

Y.O. participated in the surgical procedures, data interpretation and preparation of the manuscript.

M.T. and K.N. performed the pathological evaluation.

G.S. participated in the surgical procedures and contributed to the discussion.

H.B., R.I., K.S., Y.Shi, T.M., K.K., A.O., Y.I., H.I., T.A., and Y.T. contributed to the discussion.

All authors have read and approved the manuscript.

### Availability of data and materials

Data will be made available on reasonable request.

### Ethics approval and consent to participate

This report has been performed in accordance with the Declaration of Helsinki and was approved by the Ethical Review Board of the Cancer Institute Hospital, Japanese Foundation for Cancer Research (2023-GB-100).

### Consent for publication

Informed consent to publish has been obtained.

### Competing interests

The authors declare that they have no competing interests.

## SUPPLEMENTARY MATERIALS

Supplementary Video 1

Supplementary Video 2
